# Rectal NETs and rectosigmoid junction NETs may need to be treated differently

**DOI:** 10.1002/cam4.2779

**Published:** 2019-12-16

**Authors:** Wen Cai, Weiting Ge, Hanguang Hu, Jianshan Mao

**Affiliations:** ^1^ Department of Gastroenterology The Second Affiliated Hospital, School of Medicine, Zhejiang University Hangzhou Zhejiang China; ^2^ Cancer Institute (Key Laboratory of Cancer Prevention and Intervention, China National Ministry of Education) The Second Affiliated Hospital, School of Medicine, Zhejiang University Hangzhou Zhejiang China; ^3^ Department of Medical Oncology The Second Affiliated Hospital, School of Medicine, Zhejiang University Hangzhou Zhejiang China

**Keywords:** neuroendocrine tumors, rectosigmoid junction, rectum, risk factors

## Abstract

Neuroendocrine tumors (NETs) are heterogeneous, and the incidence of NETs is rapidly increasing. We observed different survival in patients with rectal NETs and rectosigmoid junction NETs, which are treated similarly. We included patients with rectal and rectosigmoid junction NETs from the SEER database. The 5‐year survival was set as the end‐point. 6675 patients with rectal NETs and 329 patients with rectosigmoid junction NETs, were eligible for the analysis. Initially, the survival analyses suggested that the 5‐year survival significantly differed between the patients with rectal and rectosigmoid junction NETs (HR = 0.82, 95% CI 0.70‐0.95; *P* = .01). Tumor differentiation, an invasion deeper than T2, and lymph node and distant metastases were still important risk factors affecting survival for both location. While, the males showed better survival (HR = 0.69, 95% CI 0.55‐0.88; *P* < .01) and primary tumor surgery had no benefits (*P* = .56) for patients with rectosigmoid junction NETs. The factors that predict regional lymph node metastases varied by location. In rectal NETs, invasion deeper than T1 and a tumor larger than 1 cm could significantly increase the risk of regional lymph node metastases (all OR > 5, *P* < .01). In rectosigmoid junction NETs, the risk of regional lymph node metastases was considered significantly higher with invasion deeper than T1 (all OR > 5, *P* < .01) and a tumor larger than 2 cm (OR = 31.32, 95% CI 2.53‐387.57; *P* < .01). We advocate a clear and consistent definition of the rectosigmoid junction for future studies, and more studies are needed to determine the reason underlying differences between rectum and rectosigmoid junction.

## Graphical abstract

Survival and risk factors of rectal and rectosigmoid junction NET are different. Rectosigmoid junction NETs presented a better 5‐year survival. A clear and consistent definition of the rectosigmoid junction is urgently needed for future studies.

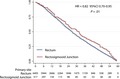

## 1 INTRODUCTION

Neuroendocrine tumors (NETs) are heterogeneous tumors with malignant potential.^1^, ^2^ The incidence has been rising, and a growing number of patients are diagnosed due to better diagnostic techniques.^3^, ^4^ The projected prevalence of NETs in the US population in 2014 was 171 321.^5^

The rectum is among the most common locations of digestive NETs (approximately 1.2/100 000 population),^6^ and the relative incidence of rectal NETs may be higher in Asian countries (approximately 50% of digestive NETs).^7^ If the disease is treated during the early stage, rectal NETs are asymptomatic and indolent, and the 5‐year survival rate is high (62%‐88%).^7^, ^8^, ^9^ However, survival is markedly worse when the disease is reginal or distant (24%‐33%).^10^, ^11^

Many different definitions have been used to divide the rectum, and its length remains controversial.^12^ Commonly, in clinical practice, we use the American Joint Committee on Cancer (AJCC) staging system, which proposes 16 cm as the upper limit of the rectum, and rectosigmoid junction tumors are defined as tumors between the sigmoid and rectum.^13^

According to the AJCC staging system, the rectosigmoid junction is a part of the rectum close to the colon, and as is well known, the overall survival of colonic NET patients is significantly worse than that of rectal NETs patients,^14^ which provoked our curiosity regarding the survival of patients with NETs located in the rectosigmoid junction. Additionally, we observed that several rectosigmoid junction NET patients shared a better survival in clinical practice, which was contrary to our thinking. The National Comprehensive Cancer Network guidelines recommend radical resection with lymph node dissection for rectal NETs >2 cm in diameter,^15^ and many studies have confirmed that the tumor size is a vital factor in predicting lymph node metastasis, which is important for deciding whether endoscopic therapies should be performed.^16^, ^17^ If patients with NETs in the rectosigmoid junction and rectum exhibit significantly different survival rates, the risk factors may also differ.

Therefore, we included patients from the Surveillance, Epidemiology, and End Results Program (SEER) database, which defined rectosigmoid junction and rectum NETs according to the AJCC staging system, to determine the survival of these two patient types and to further explore their risk factors.

## 2 MATERIALS AND METHODS

### 2.1 Data collection and patient selection

**Figure 1:**
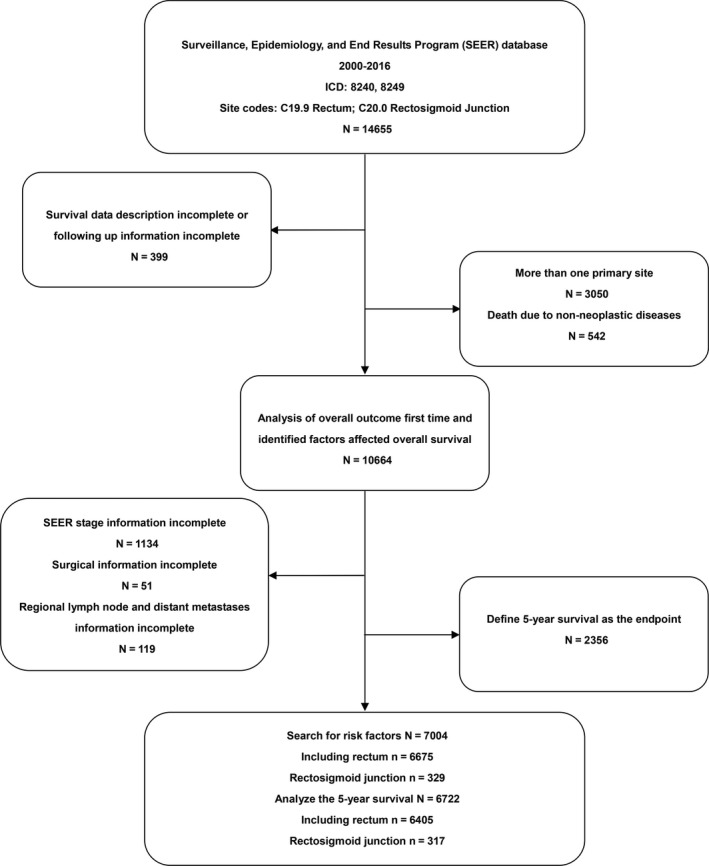
Flow chart of patient selection

The data were retrieved from the Surveillance, Epidemiology, and End Results (SEER) database based on the November 2018 submission of patients diagnosed with NETs located in the rectosigmoid junction and rectum between 2000 and 2016. We used the SEER*Stat 8.3.5 program to identify individuals in the SEER database as follows: ICD‐O‐3:8240 and 8249, and primary site codes: C19.9 rectum and C20.0 rectosigmoid junction.^15^ We excluded patients who met the following criteria: (a) patients whose survival data or follow up data were incomplete; (b) patients diagnosed with more than one primary tumor; and (c) patients whose death was due to nonneoplastic disease. Because many patients were still in the active follow up stage and the overall survival has not been achieved, we set the 5‐year survival as the end point. A flow diagram of the selection process is presented in Figure 1.

### 2.2 Definition of data

Only NETs were included in our research. The definition of rectosigmoid in the SEER database and the AJCC 8th staging system is as follows: the rectosigmoid colon joins the sigmoid colon to the rectum. The rectosigmoid is also known as the upper rectum and is generally above the peritoneal reflection.^15^ The description of the SEER staging system is as follows: localized stage (entirely confined to the organ of origin), regional stage (extending beyond the organ of origin and/or regional nodal spread), and distant stage (distant metastasis or extension).

### 2.3 Statistical analysis

The mean values are used to describe the continuous data, and the discrete variables are displayed as the totals and frequencies. The patients' demographic data and tumor characteristics are summarized using descriptive statistics. The comparisons of the categorical variables among the different groups of patients were performed using the chi‐square test. The survival function estimates and comparisons among the different variables were performed using the Kaplan‐Meier method and the log‐rank test. A Cox proportional hazards model was used to compare the effects of the prognostic variables on survival. A univariate analysis was performed using the χ^2^ test or Student *t* test. Then, a multivariable logistic regression was performed to assess the associations among the demographic/clinical factors, surgical procedure performed, and the presence of lymph node metastasis at the time of diagnosis.

All statistical analyses were performed using Intercooled Stata 12.0 (Stata Corporation). The results were considered statistically significant at a two‐sided *P* < .05.

## 3 RESULTS

### 3.1 Basic characteristics of the patients

In total, 6675 patients with rectal NETs and 329 patients with rectosigmoid junction NETs, were eligible for the analysis. The median age at diagnosis was similar (54.18‐year‐old in the rectum patients and 54.66 year‐old in the rectosigmoid junction patients). There were no significant differences in the demographic information of the patients with NETs in the two locations (sex, race, and rate of primary site surgery, all *P* > .05). Significant differences could be found in the tumor characteristics, including differentiation, the SEER stage, the TNM stage, and the tumor size, between the patients with NETs in the two locations, and these differences are summarized in Table 1. The NETs in the rectum had a smaller tumor size and were more likely to be diagnosed in the early stage than the NETs in the rectosigmoid junction. We performed multivariate analysis of including patients to identify factors affecting patients' prognosis and presented it in Table 2. The results suggested that the location of primary site (HR = 0.82, 95% CI 0.70‐0.95; *P* = .01) and TNM stage IV (HR = 2.40, 95% CI 1.30‐4.21; *P* = .01) will affect patients' prognosis. Therefore, we further investigated the relationship among tumor location, reginal or distant metastasis and patients' survival in the following.

**Table 1:** Demographic and clinical characteristics of patients

	Rectum (%)	Rectosigmoid Junction (%)	*P*‐value
Median age of diagnosis	54.18	54.66	.70
Gender			.27
Male	3273 (49.03)	151 (45.90)	
Race			.13
White	3584 (53.69)	184 (55.93)	
Black	1555 (23.30)	83 (25.23)	
Other	1536 (23.01)	62 (18.84)	
Differentiation			.02
Well	2581 (38.67)	100 (30.40)	
Moderately	423 (6.34)	21 (6.38)	
Poor	21 (0.31)	2 (0.61)	
Unreport	3650 (54.68)	206 (62.61)	
SEER stage			<.01
Localized	6491 (97.24)	304 (92.40)	
Regional	68 (1.02)	14 (4.26)	
Distant	116 (1.74)	11 (3.34)	
T stage			<.01
T1	3932 (58.91)	174 (52.89)	
T2	131 (1.96)	8 (2.43)	
T3	47 (0.7)	8 (2.43)	
T4	12 (0.18)	2 (0.61)	
Unreport	2553 (38.25)	137 (41.64)	
N stage			<.01
N0	6601 (98.89)	310 (94.22)	
N1	74 (1.11)	19 (5.78)	
M stage			.03
M0	6559 (98.26)	318 (96.66)	
M1	116 (1.74)	11 (3.34)	
Tumor size			<.01
<1 cm	3932 (58.91)	174 (52.89)	
1‐1.5 cm	131 (1.96)	8 (2.43)	
1.5‐2 cm	47 (0.70)	8 (2.43)	
>2c m	12 (0.18)	2 (0.61)	
Unreport	2553 (38.25)	137 (41.64)	
Primary site surgery			.07
Yes	5884 (88.15)	279 (84.80)	
No	791 (11.85)	50 (15.20)	

**Table 2:** Identify clinical factors association with prognosis using multivariate analysis

	HR (95% CI)	*P*‐value
Age of diagnosis	1.00 (0.99‐1.00)	.75
Gender		
Female	Reference	Reference
Male	1.01 (0.95‐1.08)	.80
Race		
White	Reference	Reference
Black	1.03 (0.95‐1.11)	.46
Other	1.05 (0.96‐1.14)	.32
Location		
Rectum	Reference	Reference
Rectosigmoid Junction	0.81 (0.69‐0.94)	.01
Differentiation		
Well	Reference	Reference
Moderately	0.91 (0.81‐1.03)	.14
Poor	1.01 (0.63‐1.63)	.96
TNM stage		
I	Reference	Reference
II	1.03 (0.81‐1.30)	.80
III	1.37 (0.97‐1.95)	.12
IV	2.40 (1.30‐4.21)	.01
Tumor size		
<1 cm	Reference	Reference
1‐1.5 cm	1.00 (0.86‐1.17)	.96
1.5‐2 cm	1.16 (0.89‐1.50)	.27
>2 cm	1.06 (0.91‐1.22)	.45

### 3.2 Survival analysis and risk factors

We set the 5‐year survival as the endpoint to perform further survival analysis because many patients were still in the follow up stage. In total, 6405 rectal NETs and 317 rectosigmoid junction NET patients were included in the analysis.

**Figure 2:**
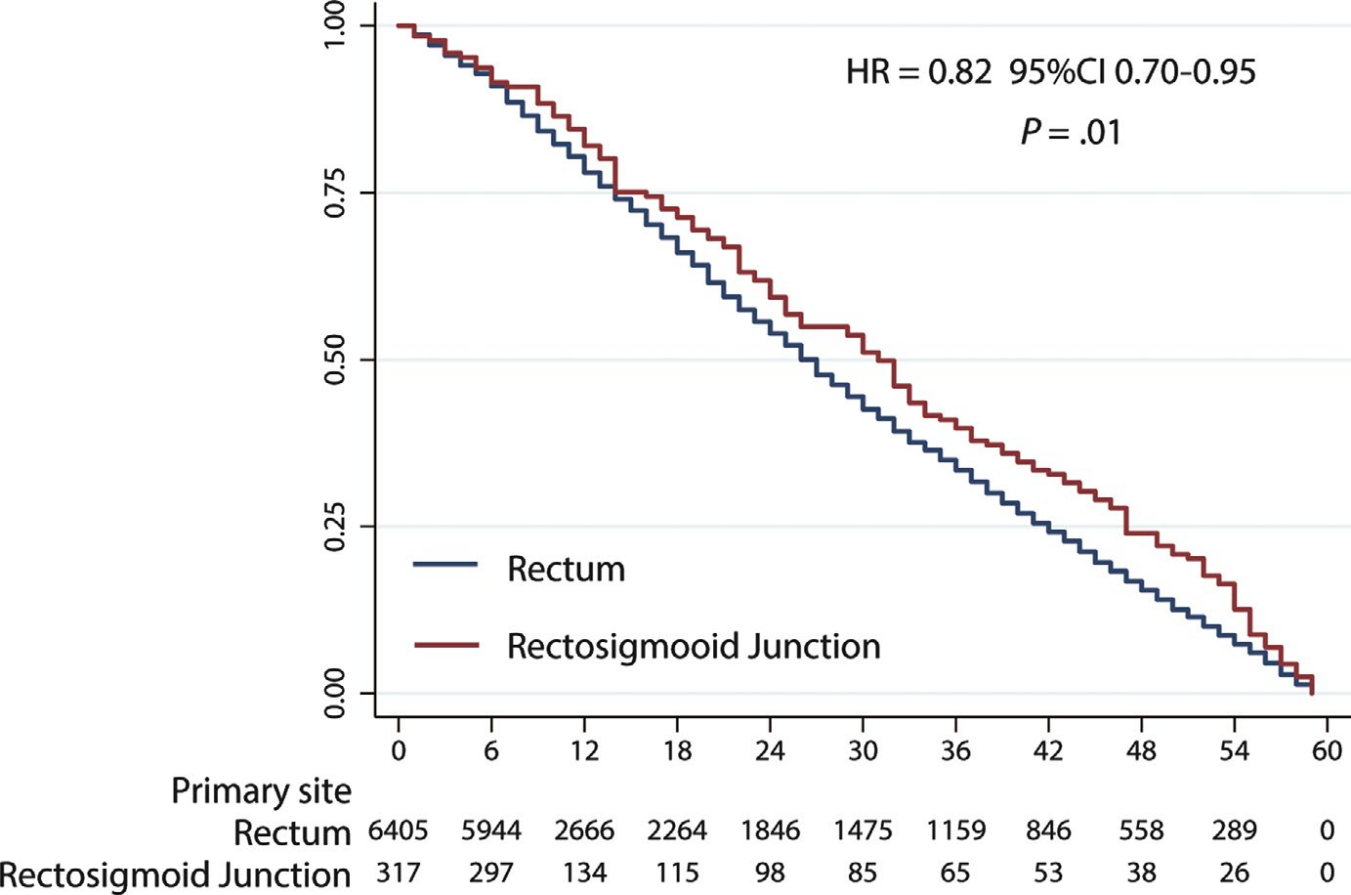
5‐year survival of rectal NETs and rectosigmoid junction is significantly different

We found that the patients with NETs in the rectosigmoid junction had a significantly better survival than those with rectal NETs (HR = 0.82, 95% CI 0.70‐0.95; *P* = .01) as shown in Figure 1 (*P* = .01, log‐rank test). We further performed a multivariate analysis of the two different locations, and the factors affecting survival differed. In the rectum, the risk factors were the same as those reported in many previous studies^18^, ^19^; poorer tumor differentiation (HR = 0.89, 95% CI 0.88‐0.90; *P* < .01; poor differentiation as the reference), deeper invasion (HR = 1.47, 95% CI 1.11‐1.95; *P* < .01; T1‐T2 as the reference), lymph node metastasis (HR = 1.29, 95% CI 1.01‐1.66; *P* = .04; N0 as the reference), and distant metastasis (HR = 11.72, 95% CI 1.41‐2.10; *P* < .01; M0 as the reference) increase the risk of tumor‐related death. The risk factors among the patients with rectosigmoid junction NETs differed such that males had a better survival than the females (HR = 0.69, 95% CI 0.55‐0.88; *P* < .01). Poor tumor differentiation (HR = 0.88, 95% CI 0.86‐0.91; *P* < .01; poor differentiation as the reference), deeper invasion (HR = 2.97, 95% CI 1.30‐6.77; *P* < .01; T1‐T2 as the reference) and distant metastasis (HR = 6.09, 95% CI 2.74‐13.56; *P* < .01; M0 as the reference) were still the main risk factors in rectosigmoid junction NETs (Figure 2). Primary surgery site could provide extra benefits to rectal NET patients (HR = 0.89, 95% CI 0.82‐0.96; *P* < .01), but the benefit was not significant in the rectosigmoid junction NET patients (HR = 0.91, 95% CI 0.65‐1.26; *P* = .56). We present these results in Table 3.

**Table 3:** Multivariate analyses of overall survival of including patients

	Rectum		Rectosigmoid Junction	
	Multivariate analyses		Multivariate analyses	
	HR (95% CI)	*P*‐value	HR (95% CI)	*P*‐value
Age of diagnosis	1.00 (0.99‐1.01)	.73	1.00 (0.99‐1.01)	.45
Gender				
Female	Reference		Reference	
Male	1.03 (0.98‐1.08)	.27	0.69 (0.55‐0.88)	<.01
Race	0.99 (0.98‐1.01)	.62	1.04 (0.98‐1.03)	.17
Differentiation	0.89 (0.88‐0.90)	<.01	0.88 (0.86‐0.91)	<.01
Tumor size	1.01 (1.00‐1.01)	.06	1.00 (0.97‐1.03)	.84
T stage				
T1‐T2	Reference		Reference	
T3‐T4	1.47 (1.11‐1.95)	<.01	2.97 (1.30‐6.77)	<.01
Unreport	1.67 (1.58‐1.76)	<.01	1.73 (1.36‐2.19)	<.01
N stage				
N0	Reference		Reference	
N1	1.29 (1.01‐1.66)	.04	1.14 (0.60‐2.16)	.68
M stage				
M0	Reference		Reference	
M1	11.72 (1.41‐2.10)	<.01	6.09 (2.74‐13.56)	<.01
Primary site surgery				
No	Reference		Reference	
Yes	0.89 (0.82‐0.96)	<.01	0.91 (0.65‐1.26)	.56

### 3.3 Different factors contribute to regional lymph node and distant metastases

Metastases definitely lead to worse survival. Therefore, we further analyzed the factors affecting regional lymph‐node and distant metastases in the patients with tumors in the two locations, and the results are summarized in Tables 4 and 5.

**Table 4:** Predictors of regional lymph nodes metastases

	Rectum		Rectosigmoid Junction	
	Multivariate analyses		Multivariate analyses	
	OR (95% CI)	*P*‐value	OR (95% CI)	*P*‐value
Age of diagnosis	0.99 (0.98‐1.01)	.41	1.03 (0.97‐1.10)	.32
Gender	1.12 (0.67‐1.87)	.66	1.83 (0.47‐7.13)	.39
Race	0.95 (0.79‐1.12)	.49	0.80 (0.48‐1.36)	.41
Differentiation	0.98 (0.91‐1.04)	.46	0.97 (0.81‐1.15)	.71
T stage				
T1	Reference		Reference	
T2	7.31 (2.97‐17.99)	<.01	16.95 (1.68‐171.04)	.02
T3	47.80 (20.30‐112.60)	<.01	35.42 (2.82‐444.51)	<.01
T4	37.22 (9.00‐153.89)	<.01	NA	NA
Unreport	2.41 (1.26‐4.61)	<.01	1.97 (0.40‐9.60)	.40
Tumor size				
<1 cm	Reference		Reference	
1‐1.5 cm	20.10 (5.36‐75.38)	<.01	6.17 (0.29‐130.54)	.24
1.5‐2 cm	66.72 (17.50‐254.36)	<.01	17.19 (0.94‐313.32)	.05
>2 cm	25.40 (7.16‐90.05)	<.01	31.32 (2.53‐387.57)	<.01
Unreport	5.62 (1.66‐19.08)	<.01	3.56 (0.37‐33.99)	.27

**Table 5:** Predictors of distant metastases

	Rectum		Rectosigmoid Junction	
	Multivariate analyses		Multivariate analyses	
	OR (95% CI)	*P*‐value	OR (95% CI)	*P*‐value
Age of diagnosis				
<54‐year‐old	Reference		Reference	
>54‐year‐old	1.03 (1.01‐1.04)	.01	1.11 (1.03‐1.21)	.01
Gender				
Female	Reference		Reference	
Male	2.03 (1.33‐3.11)	<.01	7.83 (1.16‐52.98)	.04
Race	0.88 (0.76‐1.02)	.10	1.07 (0.77‐1.48)	.69
Differentiation	1.05 (0.99‐1.07)	.07	1.00 (0.81‐1.23)	.98
Tumor size	1.01 (0.95‐1.69)	.73	1.19 (0.87‐1.62)	.27
T stage				
T1‐T2	Reference		Reference	
T3‐T4	95.85(44.87‐204.73)	<.01	2.48 (0.09‐64.83)	.59
N stage				
N0	Reference		Reference	
N1	7.75 (3.65‐16.47)	<.01	10.57 (1.03‐107.97)	<.01

After adjusting for age, sex, and race, we found that a T stage deeper than T1 and a tumor size larger than 1 cm could significantly increase the risk of regional lymph‐node metastases in patients with rectal NETs (all OR > 5.00; *P* < .01), which is consistent with many previous studies.^20^, ^21^ In the rectosigmoid junction NET patients, a T stage deeper than T1 could definitely increase the regional lymph‐node metastases risk (all OR > 5.00; *P* < .01). Furthermore, a tumor size larger than 2 cm could significantly increase the regional lymph‐node metastases risk (OR = 31.32, 95% CI 3.53‐387.57; *P* < .01), which differs from the patients with rectal NETs.

The risk factors of distant metastases slightly differed between patients with rectal NETs and rectosigmoid junction NETs. Patients older than 54 years (rectal NETs: OR = 1.03, 95% CI 1.01‐1.04; *P* < .01; rectosigmoid junction NETs: OR = 1.11, 95% CI 1.03‐1.21; *P* = .01), male patients (rectal NETs: OR = 2.03, 95% CI 1.33‐3.11; *P* < .01; rectosigmoid junction NETs: OR = 7.83, 95% CI 1.16‐52.98; *P* = .04) and regional lymph node metastases (rectal NETs: OR = 7.75, 95% CI 3.65‐16.47; *P* < .01; rectosigmoid junction NETs: OR = 10.57, 95% CI 1.03‐107.97; *P* < .01) had a higher risk of distant metastases in both locations. In rectal NETs, an invasion deeper than T2 was associated with a high risk of distant metastases (OR = 95.85, 95% CI 44.87‐204.73; *P* < .01).

## 4 DISCUSSION

In this study, we reported an unexpected result that the 5‐year survival significantly differed between patients with rectal NETs and those with rectosigmoid junction NETs. The patients with NETs in the rectosigmoid junction had a better survival than those with rectal NETs (HR = 0.82, 95% CI 0.70‐0.95; *P* = .01). We further explored the difference between the patients with rectal NETs and rectosigmoid junction NETs in many aspects. To the best of our knowledge, this study is the first to describe the difference between rectal NETs and rectosigmoid junction NETs.

At baseline, our study showed that the demographic information and surgery rate of the patients with NETs in the two locations were similar (all *P* > .05), but the characteristics of the tumors significantly differed, implying heterogeneity in the two locations. We also performed multivariate analysis of including patients which identified tumor locations and metastasis were factors affecting patients' prognosis. These results were the important basis of further analysis.

The rectosigmoid junction has been recognized as a distinct segment of the colon by the International Classification of Diseases for further heterogeneity in management and outcomes.^22^ The AJCC staging system and SEER database also separated the rectosigmoid junction, but to date, most of these tumors are treated as rectal tumors. In our study, the heterogeneities in rectosigmoid junction NETs were obvious, and we propose that future studies divide these NETs in data analyses. However, there are some opportunities and challenges. A standardized definition for the demarcation of the rectosigmoid junction is essential for further studies. However, a consensus has not been reached, which could increase the difficulties and bias in analyzing data from different countries or different data bases.^23^ The German guidelines, TNM staging and SEER staging propose 16 cm as the upper limit of the rectum, whereas 15 cm has been proposed by the United States (ASCRS), United Kingdom and European guidelines (ESMO) and the UICC Manual. Other guidelines include a distance of 12 cm (Spanish guidelines) and 9 cm (Korea).^12^, ^24^ Many studies have attempted to develop a definition because the therapeutic choice highly differs between colon and rectal adenocarcinoma.^25^, ^26^, ^27^ If a rectal tumor is misclassified as a sigmoid tumor, the patient could be inadequately staged and not considered for preoperative downstaging (chemo) radiation, potentially decreasing their chance of undergoing a complete resection and worsening their survival.^27^, ^28^, ^29^ Due to the heterogeneities of NETs, a proper definition and the separation of these two types of tumors could be more meaningful and useful.

The factors affecting overall survival in patients with rectal NETs and rectosigmoid junction NETs also differed. In addition to the common factors, such as tumor differentiation, T stage and M stage, males had a better survival than females (HR = 0.69, 95% CI 0.55‐0.88; *P* < .01), and the benefits of primary surgery were not significant (HR = 0.91, 95% CI 0.65‐1.26; *P* = .56) in rectosigmoid junction NETs. As is well known, surgery is not the only way to remove tumors in the rectum.^30^ The optimal ways for primary resection in rectal NETs still remain controversial. Endoscopic resection has been shown to be effective in removing rectal NETs, particularly those measuring <10 mm in size.^7^ However, the treatment choices still vary because sufficient and convictive data are lacking, rendering it difficult to ensure complete tumor resection and lower the rate of recurrence.^15^ The North American Neuroendocrine Tumors Society guidelines conclude that tumors <2 cm that are confined to the mucosa or submucosa are associated with very minimal risk of local and metastatic spread, and metastatic screening or follow‐up are not recommended after local resection.^7^ In contrast, the National Comprehensive Cancer Network guidelines suggest that all patients should be screened with colonoscopy plus either abdominal/pelvic CT/MRI and endorectal ultrasound or endoscopic ultrasound.^15^ In addition, for lesions ≤2 cm, the National Comprehensive Cancer Network suggests trans‐anal excision if possible with no follow‐up for lesions <1 cm and follow‐up at 6 and 12 months for local recurrence with rectal MRI or endoscopic ultrasound for lesions between 1 and 2 cm.^15^ In practice, most lesions <2 cm are endoscopically resected without lymph node harvest. However, as described in the recent European Neuroendocrine Tumor Society consensus update for neuroendocrine neoplasms, additional studies are still needed to determine whether local resections are indeed sufficient for preventing recurrence.^14^

Based on the above, we can infer that that controversy regarding endoscopic or surgical resection focuses on the tumor size and lymph node metastases rather than critical factors associated with recurrence.^31^, ^32^, ^33^ In our study, we performed multivariate analyses to identify the different risk factors contributing to lymph node and distant metastases. Our results concerning rectal NETs were consistent with previous studies,^33^ that is, a T stage deeper than T1 and a tumor size larger than 1 cm significantly increase the risk of regional lymph‐node metastases, and patients older than 54 years, male patients, patients with an invasion deeper than T2 and patients with regional lymph node metastases have a higher risk of distant metastases.

In rectosigmoid junction NETs, some risk factors have never been previously reported to be associated with recurrence, and we recommend that in addition to a T stage deeper than T1, a tumor size larger than 2 cm could significantly increase the risk of regional lymph‐node metastases and distant metastases.

Considering the above, we advocate for a clearer and more consistent definition of the rectosigmoid junction and to investigate it separately from rectal NETs in future studies. Future studies investigating rectal NETs can also divide into different lengths, which may provide more information to determine NETs' heterogeneities.

## 5 CONCLUSION

Patients with rectosigmoid junction NETs have a better survival and different risk factors than those with rectal NETs. The treatment choices for rectal NETs and rectosigmoid junction NETs may need to be reconsidered. A clear and consistent definition of the rectosigmoid junction is urgently needed for future further studies, and more studies are needed to determine the reason underlying these differences.

## CONFLICT OF INTEREST

The authors have no conflicts of interest to declare.

## AUTHOR CONTRIBUTIONS

All authors contributed to the study conception and design. Material preparation, data collection and analysis were performed by Wen Cai and Weiting Ge. The first draft of the manuscript was written by Wen Cai. Jianshan Mao and Hanguang Hu check the data and all authors commented on previous versions of the manuscript. All authors read and approved the final manuscript.
